# Evans Syndrome

**DOI:** 10.5811/cpcem.2019.1.41028

**Published:** 2019-02-26

**Authors:** Ahmed Al Hazmi, Michael E. Winters

**Affiliations:** * University of Maryland Medical Center, Department of Emergency Medicine, Baltimore, Maryland; † University of Maryland School of Medicine, Department of Emergency Medicine, Baltimore, Maryland

## Abstract

A 22-year-old man presented to the emergency department with facial swelling, rash, and fatigue. He had a past medical history of pericarditis and pericardial effusion. His evaluation showed anemia and thrombocytopenia. He was admitted for intravenous administration of steroids, plasmapheresis, and workup of his anemia and thrombocytopenia. He was ultimately diagnosed with Evans syndrome as a presenting feature of systemic lupus erythematosus. Plasmapheresis was stopped but administration of steroids continued. His blood counts improved, and the facial swelling and rash subsided. Evans syndrome is an immunologic conundrum that requires early recognition and treatment.

## INTRODUCTION

Evans syndrome (ES) is a very rare autoimmune disease first described in 1951. It is the combination of Coombs-positive idiopathic autoimmune hemolytic anemia (IAHA) and immune thrombocytopenic purpura (ITP).^1^–^3^ In addition, ES can be associated with the development of neutropenia due to autoimmune destruction. Though the degree of immunosuppression can be profound, there are no reported cases of ES patients with neutropenia experiencing life-threatening infections.^2^,^4^,^5^ Importantly, IAHA, ITP, and neutropenia can develop sequentially or can all be present at the time of diagnosis. Often, patients with ES have discordance between their clinical symptoms and the severity of their laboratory abnormalities.^6^,^7^ ES is a chronic autoimmune condition characterized by exacerbations and periods of remission.^1^–^3^ Patients who are experiencing an exacerbation often present to the emergency department (ED) for evaluation and management. To minimize morbidity and the risk of death, it is important for the emergency physician to identify patients with ES and institute urgent therapy. In this case report, we describe an atypical presentation of ES in a young man who presented to our ED.

## CASE REPORT

A 22-year-old male with a past medical history of pericarditis and pericardial effusion presented to the ED with the chief complaint of facial swelling, which had been present for the prior three weeks. The swelling was predominantly on the right side of his face and upper lip. He had no history of angioedema, had not started any new medications, and was not aware of an environmental exposure that immediately preceded the onset of swelling. In addition to the facial and lip swelling, the patient reported a rash of the same duration on his chest and shoulders. Additional associated symptoms included decreased exercise tolerance, exertional dyspnea, and a single episode of dark, maroon-colored stool. He denied fever, chills, myalgia, arthralgia, chest pain, abdominal pain, nausea, vomiting, odynophagia, dysphagia, and confusion. He was not aware of any sick contacts and he had not traveled recently. He reported that his family did not have a history of chronic illnesses.

**Image 1:**
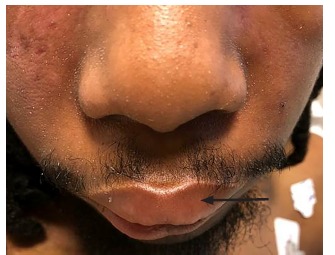
Bilateral facial edema along with edema of the upper lip (arrow).

**Image 2:**
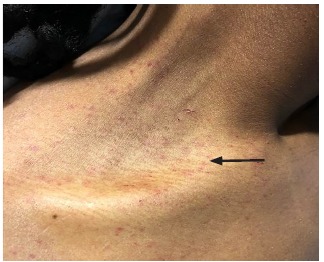
Petechial rash (arrow) on upper chest and shoulders.

**Image 3:**
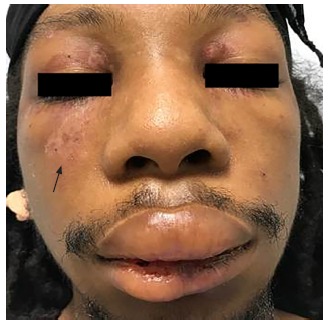
Malar rash (arrow) in addition to facial and lip swelling.

Physical examination was significant for a blood pressure of 104/58 millimeters of mercury, a pulse of 96 beats per minute, respiratory rate of 16 breaths per minute, a temperature of 36.8° Celsius, and a pulse oximetry reading of 100% on room air. He was a thin young man who did not appear to be in distress or acutely ill. Bilateral facial edema along with edema of the upper lip was noted (Image 1). In addition, his conjunctiva, palms, and soles were notable for pallor. A petechial rash was observed on his upper chest, bilateral shoulders, tongue, and soft palate (Image 2). A malar rash was also noted (Image 3). The remainder of his examination was normal.

His initial ED evaluation included a chest radiograph, electrocardiogram, and laboratory studies. The results of pertinent laboratory studies are listed in the table. Given his severe thrombocytopenia and anemia, thrombotic thrombocytopenic purpura (TTP) was considered and an emergent hematology consultation was obtained. A peripheral blood smear demonstrated 1–2 schistocytes per high-power field, which initially raised concern for a microangiopathic hemolytic anemia. As a result, a hemodialysis catheter was inserted and plasmapheresis was initiated while the patient was in the ED. He received a unit of packed red blood cells along with corticosteroids and was admitted to the medical intermediate care unit.

**CPC-EM Capsule**

What do we already know about this clinical entity?

*Evans syndrome is a rare autoimmune disease characterized by autoimmune hemolytic anemia and immune thrombocytopenic purpura*.

What makes this presentation of disease reportable?

*To our knowledge, this is the first case report to detail the clinical presentation of a patient with Evans syndrome to the emergency department. The patient presented with angioedema, fatigue, and a petechial rash*.

What is the major learning point?

*Evans syndrome often presents with features of other autoimmune disorders and can frequently be misdiagnosed*.

How might this improve emergency medicine practice?

*In addition to thrombotic thrombocytopenic purpura, emergency physicians should consider the diagnosis of Evans syndrome in patients presenting with thrombocytopenia and a hemolytic anemia*.

Workup revealed a positive immunoglobulin G (IgG) Coombs test. He also had a high titer of antinuclear acid antibody and low C3/C4 complements, indicative of an acute exacerbation of an autoimmune disease. The combination of his symptoms, ED workup, and history of pericarditis and pericardial effusion favored the diagnosis of systemic lupus erythematosus (SLE). Within 48 hours after admission, an A disintegrin and metalloproteinase with thrombospondin motifs 13 (ADAMTS13) level returned with 78% activity and less than 5% inhibitor. This result was not consistent with the diagnosis of TTP, and plasma exchange was stopped. Ultimately, the hematologist diagnosed Evans syndrome as a presenting feature of SLE.

## DISCUSSION

ES is a rare autoimmune disorder characterized by profound immune dysregulation. To date, no cause has been identified and the pathophysiology remains uncertain.^2^ Notwithstanding, ES has been associated with select infections and both the measles-mumps-rubella and influenza vaccines.^4^,^8^ It is hypothesized that immunization can trigger ES in susceptible individuals.^4^,^8^

ES presents with signs and symptoms of both IAHA and ITP. Typical symptoms include fatigue, pallor, dyspnea, tachycardia, and fever. Jaundice, hematuria, hemoglobinuria, and hepatosplenomegaly might also be noticed. Patients with ES could have bruising, petechiae, and mucocutaneous bleeds secondary to thrombocytopenia. It is important to also note that patients may have neutropenia and may present with immunosuppression-related infections. The severity of the disease can vary from mild to life-threatening.^9^

Importantly, ES is a diagnosis of exclusion. Although the hallmark laboratory abnormalities point to IAHA and ITP, no single test can confirm the diagnosis of ES. In fact, many of its laboratory abnormalities are also seen in other conditions with similar clinical presentations (e.g., low level schistocytes). These include other autoimmune conditions (e.g., SLE), IgA deficiency, TTP, autoimmune myeloproliferative syndromes, and malignancy. Further complicating the diagnostic process is the fact that autoimmune destruction of circulating blood cells in autoimmune hemolytic anemia (AIHA) and ITP are also seen simultaneously in patients with paroxysmal nocturnal hematuria, hemolytic uremic syndrome, and hemangiomas with thrombocytopenia.^10^

Laboratory studies that are helpful in the management and workup are a complete blood count that shows pancytopenia and a peripheral blood smear that shows features of AIHA (spherocytosis). These findings will differentiate AIHA from myelodysplastic syndromes, microangiopathic hemolytic anemias, congenital anemia, and thrombocytopenia. Other markers that can be used to identify hemolysis are an elevated reticulocyte count, the presence of unconjugated hyperbilirubinemia, and a decreased haptoglobin level. Additionally, the direct Coombs test is invariably positive in patients with ES.^11^

Patients with an initial diagnosis of ES or an exacerbation of known disease should be treated with high-dose corticosteroids. In most cases, prednisone in a dose of 1–2 milligrams per kilogram is adequate to induce remission. Unfortunately, a subset of patients with ES is recalcitrant to this approach, and some have varied responses to the intravenous administration of immunoglobulin (IVIG). In addition, responses to the IVIG range from remission to no effect. Ultimately, patients who are refractory to corticosteroids and IVIG might require advanced therapies such as monoclonal antibodies (rituximab), cyclosporine, and splenectomy with stem cell transplant. It is imperative to avoid administering blood products to those patients unless they have profound anemia or life-threatening bleeding.^12^

## CONCLUSION

Emergency physicians should consider ES in patients with features of a hemolytic anemia and thrombocytopenia. Early recognition and treatment can reduce the morbidity and mortality associated with this immunologic conundrum.

## Figures and Tables

**Table:** Laboratory results relevant for Evans syndrome in the emergency department.

WBC	3.4 K/mcL
RBC	1.81 M/mcL
Hb	5.8 g/dL
HCT	17.2 %
PLT	4 K/mcL
LDH	389 units/L
AST	437 units/L
ALT	117 units/L
Total bilirubin	4.4 mg/dL
Indirect bilirubin	0.6 mg/dL
INR	1.2
PTT	42 seconds

*WBC*, white blood cells; *K*, thousands; *mcL*, microliter; *RBC*, red blood cells; *M*, moles; *Hb*, hemoglobin; *g/dL*, grams per deciliter; *HCT*, hematocrit; *PLT*, platelet; *LDH*, lactic dehydrogenase; *L*, liter; *AST*, aspartate aminotransferase; *ALT*, alanine aminotransferase; *mg*, milligrams; *INR*, international normalized ratio; *PTT*, partial thromboplastin time.

## References

[1] Evans RS, Takahashi K, Duane RT (1951). Primary thrombocytopenic purpura and acquired hemolytic anemia; evidence for a common etiology. AMA Arch Intern Med.

[2] Wang W, Herrod H, Pui CH (1983). Immunoregulatory abnormalities in Evans syndrome. Am J Hematol.

[3] Evans RS, Duane RT (1949). Acquired hemolytic anemia; the relation of erythrocyte antibody production to activity of the disease; the significance of thrombocytopenia and leukopenia. Blood.

[4] Shlamovitz GZ, Johar S (2013). A case of Evans’ syndrome following influenza vaccine. J Emerg Med.

[5] Savaşan S, Warrier I, Ravindranath Y (1997). The spectrum of Evans’ syndrome. Arch Dis Child.

[6] Sarode R (2009). Atypical presentations of thrombotic thrombocytopenic purpura: a review. J Clin Apher.

[7] Zhang C, Chen XH, Zhang X (2014). Quick development and sudden death: Evans syndrome followed by thrombotic thrombocytopenic purpura. Am J Emerg Med.

[8] Wucherpfennig KW (2001). Mechanisms for the induction of autoimmunity by infectious agents. J Clin Invest.

[9] Miano M (2016). How I manage Evans syndrome and AIHA cases in children. Br J Haematol.

[10] Akbary S, Kannikeswaran N (2012). Acute onset altered mental status in a previously healthy teenager. Pediatr Emerg Care.

[11] Norton A, Roberts I (2006). Management of Evans syndrome. Br J Haematol.

[12] Hill QA, Stamps R, Massey E (2017). Guidelines on the management of drug-induced immune and secondary autoimmune, haemolytic anaemia. Br J Haematol.

